# Long Non-Coding RNA *StlincPRI* Enhances Resistance to *Phytophthora infestans* by Regulating Reactive Oxygen Species Homeostasis

**DOI:** 10.3390/plants15203191

**Published:** 2026-10-18

**Authors:** Rui Lin, Na Zhou, Jiaxin Hao, You Wu, Wei Bai

**Affiliations:** College of Life Sciences, Inner Mongolia Agricultural University, Hohhot 010018, China

**Keywords:** potato, lncRNA, late blight, plant immune response, oxidative damage

## Abstract

Potato (*Solanum tuberosum*) is the fourth-largest food crop worldwide. However, late blight, caused by oomycete *Phytophthora infestans*, poses a major threat to global production. Although long non-coding RNAs (lncRNAs) have emerged as key regulators of plant immune responses, their role in potato immune response remains largely unknown. Here, transcriptome profiling of resistant cultivar Qingshu No. 9 and susceptible cultivar Désirée identified a *P. infestans*-responsive long intergenic non-coding RNA (lincRNA), named *StlincPRI*, which is significantly upregulated upon infection. Overexpression of *StlincPRI* activates the expression of pathogenesis-related genes, inhibits pathogen growth, and decreases lesion area upon *P. infestans* challenge. Moreover, *StlincPRI* triggers a rapid reactive oxygen species (ROS) burst during the early stage of infection. Subsequently, it alleviates oxidative damage via enhancement of both enzymatic and non-enzymatic ROS scavenging systems to maintain cellular ROS homeostasis. Conversely, silencing *StlincPRI* results in opposite effects, demonstrating that *StlincPRI* functions as a positive regulator of potato immunity against *P. infestans*. These findings provide insights for developing strategies to enhance late blight resistance and hold potential for advancing sustainable potato production.

## 1. Introduction

Potato ranks as the fourth most important food crop globally after wheat, rice and maize. Owing to its high nutritional value and substantial starch yield, potato is an important staple food for human consumption. According to FAOSTAT, the official statistical database of the Food and Agriculture Organization of the United Nations (FAO), global potato production reached approximately 390.4 million tonnes in 2024. Potato is not only a key component of global food security systems but also a versatile biomass resource with various exploration potential. However, yield and quality of potato are severely threatened by the oomycete pathogen *Phytophthora infestans*, which is the causal agent of late blight disease. For instance, potato late blight contributed to the Irish Great Famine in the 1840s. Famine-related mortality and mass emigration contributed to a nearly 20% decline in Ireland’s population between 1841 and 1851 [1]. The control of late blight has been a major challenge in agriculture for a long time. Traditional strategies rely heavily on chemical fungicides which lead to health risks, environmental pollution, and the emergence of fungicide-resistant pathogens. Therefore, breeding resistant cultivars has become a safer and more sustainable strategy for disease management [2]. A key step in resistance breeding is the identification and utilization of resistance gene resources. Wild species of *Solanum* harbor abundant resistance genes, and to date, more than 70 resistance genes have been identified [3]. However, introducing these resistant traits into elite cultivars remains challenging. Traditional hybrid breeding is time-consuming and the entire process often takes over a decade to complete. With the advancement of high-throughput sequencing and functional genomics, an increasing number of genes responsible for innate immunity have been characterized and exploited as useful genetic resources for subsequent plant breeding [4].

Historically, research on the immune response in plants has primarily focused on protein-coding genes. The roles of these genes in plant defense mechanisms have been extensively and systematically investigated. However, the dynamic and multilayered nature of plant–pathogen interactions indicates that additional genomic components, including non-coding RNAs (ncRNAs), also participate in the regulation of host immunity. NcRNAs represent a considerable fraction of the eukaryotic transcriptome and are increasingly recognized as functional molecules involved in diverse biological processes. Among these, lncRNAs, generally defined as transcripts longer than 200 nucleotides without apparent protein-coding potential, have emerged as important regulators. In particular, lincRNAs, which originate from intergenic genomic regions, have been implicated in plant development as well as responses to biotic and abiotic stresses. Genome-wide identification of differentially expressed lincRNAs (DE-lincRNAs) was conducted in grafted pepper. It suggested that DE-lincRNAs increased the resistance of grafted pepper to *P. capsici* by cis-regulating the expression of disease-defense related genes [5]. In *Arabidopsis thaliana*, the elf18-induced lincRNA *ELENA1* enhances resistance to *Pseudomonas syringae* pv. *tomato* DC3000 by directly interacting with mediator complex subunit MED19a and promoting its enrichment at the *PR1* promoter [6]. In *Solanum tuberosum*, transcriptome sequencing identified a set of lincRNAs responsive to *‘Candidatus* Liberibacter’ infection. These lincRNAs predominantly act in cis to regulate the expression of adjacent protein-coding genes. Functional characterization of selected lincRNAs that were differentially expressed upon psyllid-mediated ‘*Ca.* L. solanacearum’ infection revealed that their overexpression reduced pathogen accumulation and enhanced host tolerance [7]. *SUNA1*, a salicylic acid (SA)-activated lincRNA, positively regulates *Arabidopsis* disease resistance by modulating precursor ribosomal RNA (pre-rRNA) processing and translational efficiency of defense genes [8]. Transcriptome profiling of *Solanum lycopersicum* following *Ralstonia solanacearum* inoculation identified 315 lincRNAs, of which 23 were differentially expressed during infection. These responsive lincRNAs may act in cis to regulate adjacent genes associated with fatty acid elongation, jasmonic acid and ethylene signaling pathways, as well as ARGONAUTE (AGO)-related regulatory processes, thereby contributing to tomato responses to bacterial wilt [9]. However, investigations addressing the functions of lincRNAs in potato resistance to *P. infestans* remain limited. Recent studies have shown that a comprehensive integration of 364 RNA sequencing (RNA-seq) datasets across 12 biotic stress conditions has enabled systematic identification of stress-responsive lncRNAs in potato [10]. In another case, analysis of thirty-six late blight–related transcriptomes revealed 2857 lncRNAs responsive to *P. infestans*, highlighting the extensive involvement of non-coding transcripts in disease defense [11]. Moreover, *StlncRNA13558* is an abscisic acid (ABA)-inducible lncRNA that functions in cis to modulate downstream resistance gene *StPRL*, enhancing potato resistance to *P. infestans* [12].

In this study, *StlincPRI*, a differentially expressed lincRNA responsive to *P. infestans* infection, was identified. Functional analyses revealed that *StlincPRI* enhances resistance to *P. infestans* by modulating reactive oxygen species homeostasis and upregulating expression of PR genes. These findings enrich the understanding of the molecular mechanisms underlying potato disease resistance and provide a foundation for future strategies to leverage plant innate immunity to improve resistance.

## 2. Results

### 2.1. Time-Series Transcriptomic Profiling Identifies a Core Candidate Gene from the Union of Differentially Expressed Genes

**Figure 1:**
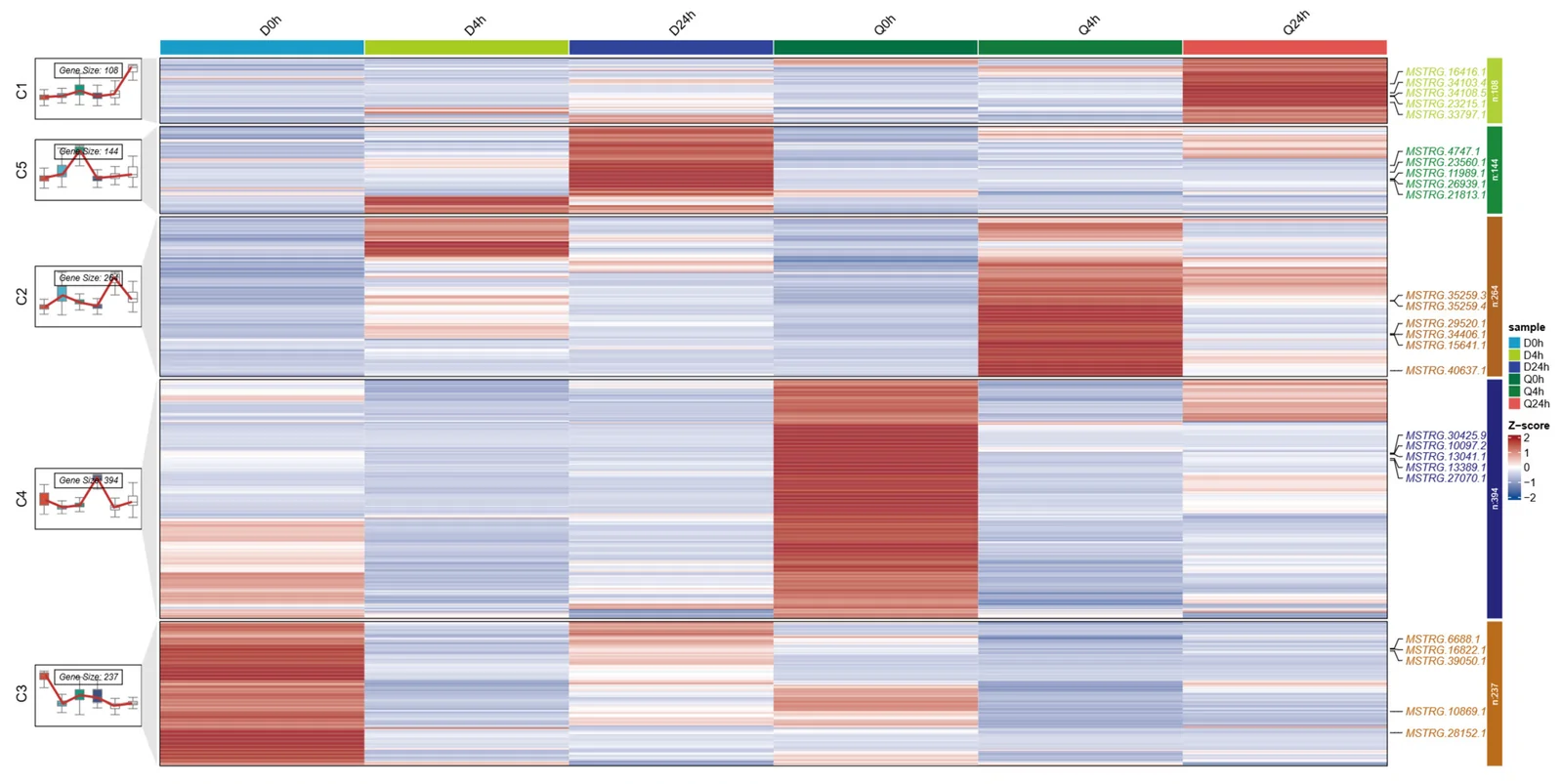
Time-series expression clustering of differentially expressed genes. Q 0 hpi: pre-inoculation samples of cultivar Qingshu No. 9. Q 4 hpi: 4 h post-infection with *P. infestans* in cultivar Qingshu No. 9. Q 24 hpi: 24 h post-infection with *P. infestans* in cultivar Qingshu No. 9. D 0 hpi: Pre-inoculation samples of cultivar Désirée. D 4 hpi: 4 h post-infection with *P. infestans* in cultivar Désirée. D 24 hpi: 24 h post-infection with *P. infestans* in cultivar Désirée.

To investigate the transcriptional dynamics of potato in response to *P. infestans*, we compared transcriptome profiles of the resistant cultivar Qingshu No. 9 (Q) and the susceptible cultivar Désirée (D) at 0, 4 and 24 h post-inoculation (hpi). Differentially expressed genes (DEGs) were identified in four pairwise comparisons (D4 hpi vs. D0 hpi, D24 hpi vs. D0 hpi, Q4 hpi vs. Q0 hpi and Q24 hpi vs. Q0 hpi) using thresholds of |log_2_FC| > 1 and *p* < 0.05. The overall distribution and expression patterns of the DEGs were visualized using volcano plots and heatmaps, respectively (Figure S1). Subsequently, the union of DEGs from the four comparisons was analyzed across three sampling time points (0, 4, and 24 hpi) in each cultivar to characterize temporal expression patterns. This approach grouped the DEGs into 5 distinct clusters based on their expression profiles over time (Figure 1). For example, cluster 2 genes exhibited a unique and highly consistent expression pattern, characterized by sharp upregulation at an early time point followed by progressive decline in both cultivars from Désirée and Qingshu No. 9 backgrounds. Notably, the magnitude of this upregulation was greater in cultivar Qingshu No. 9 than in cultivar Désirée, suggesting their potential role as key regulators of *P. infestans* resistance.

The cluster 2 correlation table contained 33 transcript-pair records involving 38 distinct transcripts. Among these, 22 nonredundant transcripts listed in the parameter1 field had complete expression profiles across the six conditions together with corresponding cluster membership values and were therefore included in the downstream candidate-prioritization analysis (Table S4). Using a template-similarity threshold of *s_i_* > 0.99, six candidate transcripts were retained: MSTRG.40637.1, MSTRG.28159.1, MSTRG.31972.1, MSTRG.27755.1, MSTRG.17008.2 and MSTRG.28780.6. Among these, MSTRG.40637.1 showed a strong match to the idealized expression pattern characterized by a peak at 4 hpi in the resistant cultivar Qingshu No. 9, with a template correlation coefficient of 0.995057. Its cluster membership value was 0.895401, the highest among the six retained candidates, and it was therefore prioritized for subsequent functional investigation. The standardized expression values of MSTRG.40637.1 were −0.472897, −0.400606, −0.469321, −0.472897, 2.031152 and −0.215430 at D0 hpi, D4 hpi, D24 hpi, Q0 hpi, Q4 hpi and Q24 hpi, respectively. This expression profile was characterized by a pronounced peak at 4 hpi in cultivar Qingshu No. 9, followed by a decline at 24 hpi, whereas relatively little variation was observed across the three time points in cultivar Désirée (Figure S2). In the transcript-pair correlation analysis, MSTRG.40637.1 also showed a strong positive correlation with MSTRG.7593.1 across the six conditions (*r* = 0.999493), further supporting the concordance of their expression patterns. Sensitivity analysis showed that the same six candidates were retained when the template-similarity threshold was set at 0.985, 0.990 or 0.995, with MSTRG.40637.1 consistently exhibiting the highest cluster membership value. At a threshold of 0.980, MSTRG.31606.2 showed the highest membership value among the retained candidates, whereas MSTRG.40637.1 was excluded when the threshold was increased to 0.996. Thus, the prioritization of MSTRG.40637.1 was robust across similarity thresholds ranging from 0.985 to 0.995, although its selection remained dependent on the requirement for close agreement with the 4 hpi peak-expression pattern in cultivar Qingshu No. 9. Taken together, these expression characteristics and prioritization criteria supported the selection of MSTRG.40637.1 as the leading candidate from cluster 2 for subsequent functional analysis. MSTRG.40637.1 was annotated as a 456-nt, two-exon transcript on the negative strand of chromosome 12, spanning positions 54,954,256–54,955,091. The original transcript annotation assigned it class code “u”, indicating an intergenic transcript, and placed it between Soltu.DM.12G024930.1 and Soltu.DM.12G024940.1 (Figure S3). Its original Coding-Non-Coding Index (CNCI) score was −0.053248, and additional Coding Potential Calculator 2 (CPC2) analysis classified the verified 456-nt sequence as non-coding, with a coding probability score of 0.11424 (Table S3). These findings support its annotation as a predicted lincRNA, which was designated *StlincPRI* (positive regulator of immunity).

### 2.2. StlincPRI Is Upregulated in Response to P. infestans Infection

**Figure 2:**
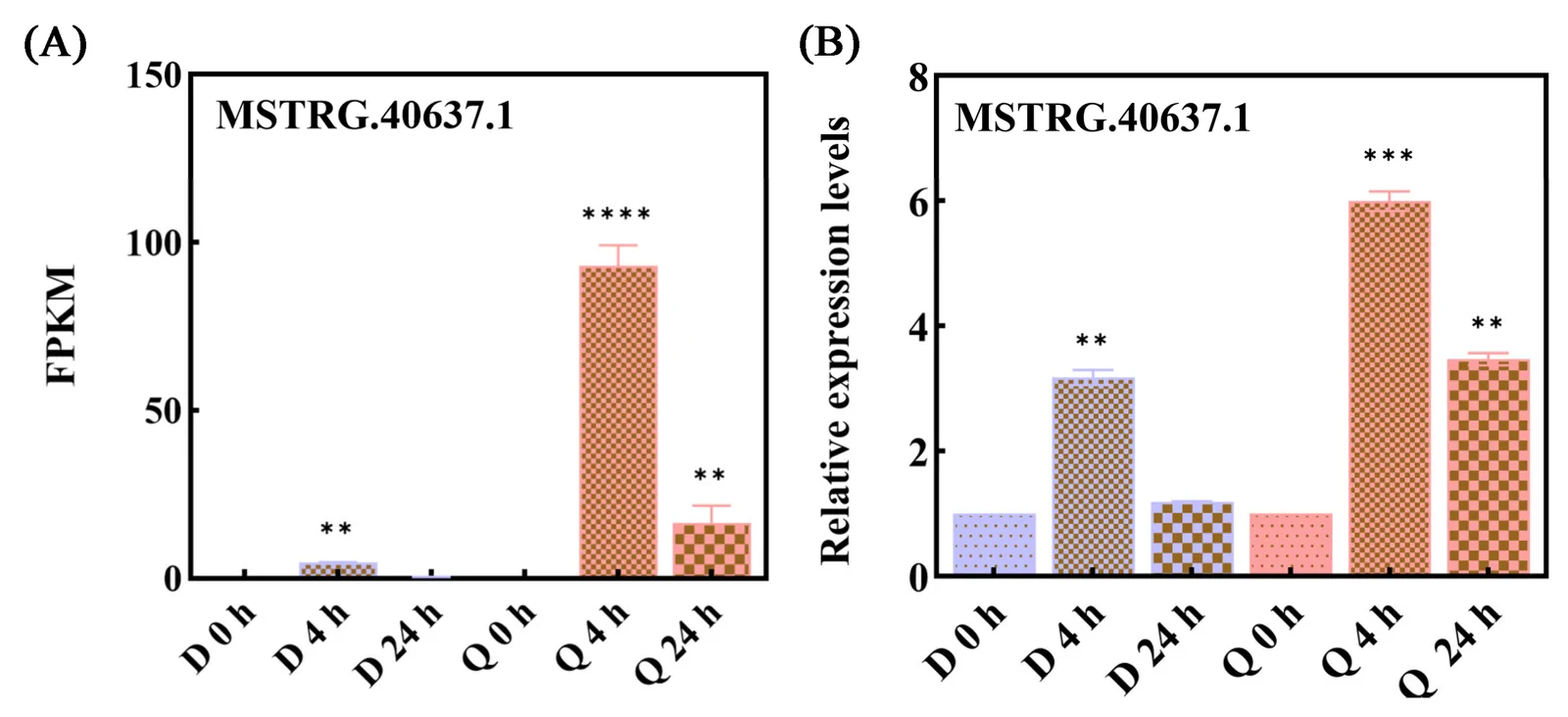
Expression analysis of *StlincPRI* following *P. infestans* infection. (**A**) Expression levels of *StlincPRI* in the transcriptome. (**B**) Validation of *StlincPRI* transcriptome data through RT-qPCR. Data are presented as mean ± standard deviation (SD) (*n* = 3). Significant differences were assessed using Student’s *t*-test by comparing each post-inoculation time point with the corresponding 0 h group within the same cultivar; ** *p* < 0.01; *** *p* < 0.001; **** *p* < 0.0001.

The expression level of *StlincPRI* increased at 4 h and subsequently decreased at 24 h in both the susceptible and the resistant cultivar in transcriptome data. Moreover, its expression level was much higher in the resistant cultivar than in the susceptible one (Figure 2A). It was then confirmed the *StlincPRI* expression pattern by reverse transcription quantitative PCR (RT-qPCR) assay and found that its expression pattern is consistent with the results obtained from transcriptome analysis (Figure 2B).

### 2.3. StlincPRI Enhances Resistance to P. infestans in Nicotiana benthamiana

**Figure 3:**
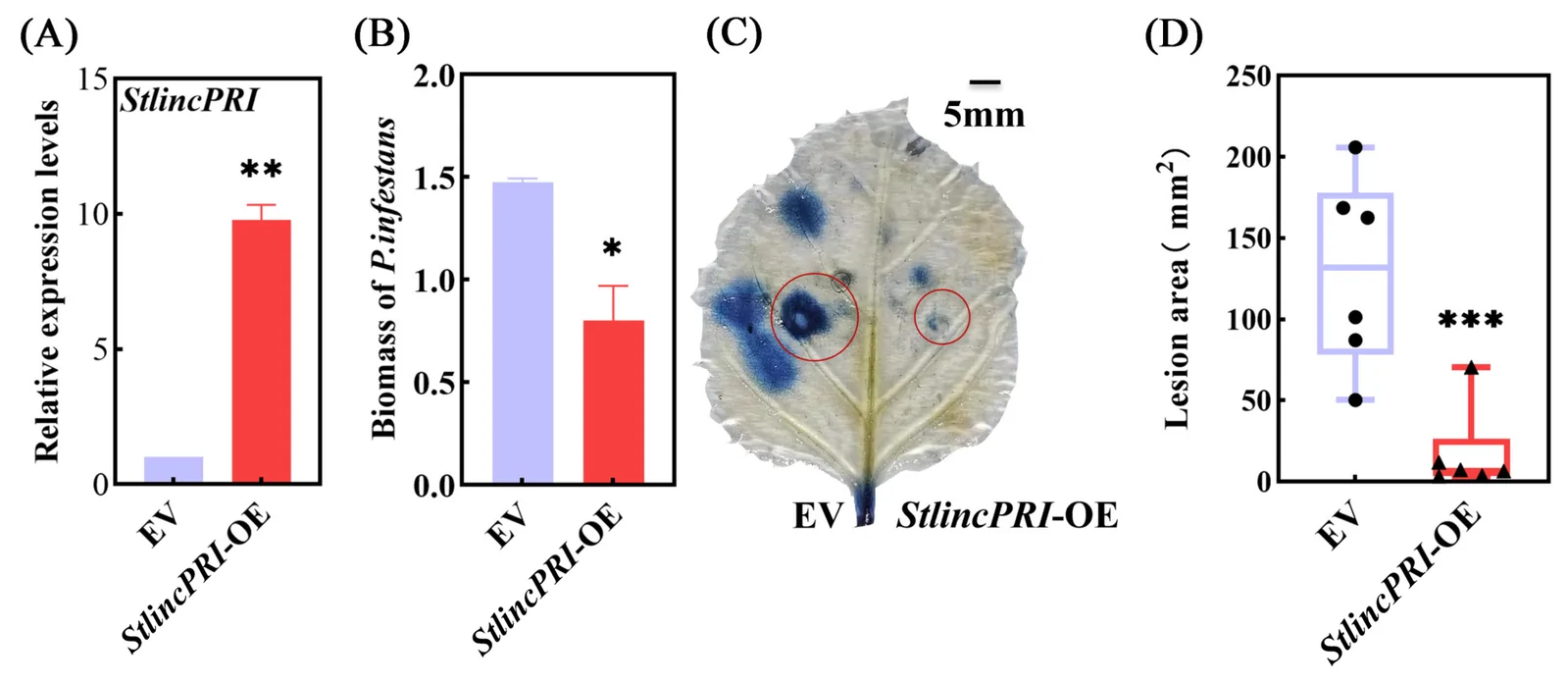
Effects of *StlincPRI* overexpression on tobacco resistance to *P. infestans*. (**A**) Relative expression levels of *StlincPRI* following transient transformation. (**B**) Relative *P. infestans* biomass estimated by qPCR targeting the *P. infestans*-specific element *PiO8*. (**C**) Representative trypan blue-stained detached leaf, with lesions outlined. Scale bar = 5 mm. (**D**) Quantification of lesion area. Boxes represent the 25th–75th percentiles, centre lines indicate the medians, and whiskers extend to the minimum and maximum values. Each point represents one leaf. Six leaves from three independent plants were evaluated per treatment; leaves from the same plant were subsamples. EV, empty vector control; *StlincPRI*-OE, *StlincPRI* overexpression. Data in (**A**,**B**) are presented as mean ± SD (*n* = 3). Statistical comparisons were performed between the overexpression treatment and its corresponding EV control using Student’s *t*-test. * *p* < 0.05; ** *p* < 0.01; *** *p* < 0.001.

The role of *StlincPRI* in detached tobacco leaves against *P. infestans was examined*. At 48 h after transient transformation, *StlincPRI* overexpression (*StlincPRI*-OE) *N. benthamiana* leaves showed *StlincPRI* RNA levels 8.3-fold higher than control leaves (Figure 3A). It was then inoculated these tobacco leaves with *P. infestans* and found that *StlincPRI*-OE leaves displayed 0.7-fold pathogen biomass and 0.1-fold lesion area compared with control (Figure 3B–D).

### 2.4. StlincPRI Enhances Resistance to P. infestans in Potato

**Figure 4:**
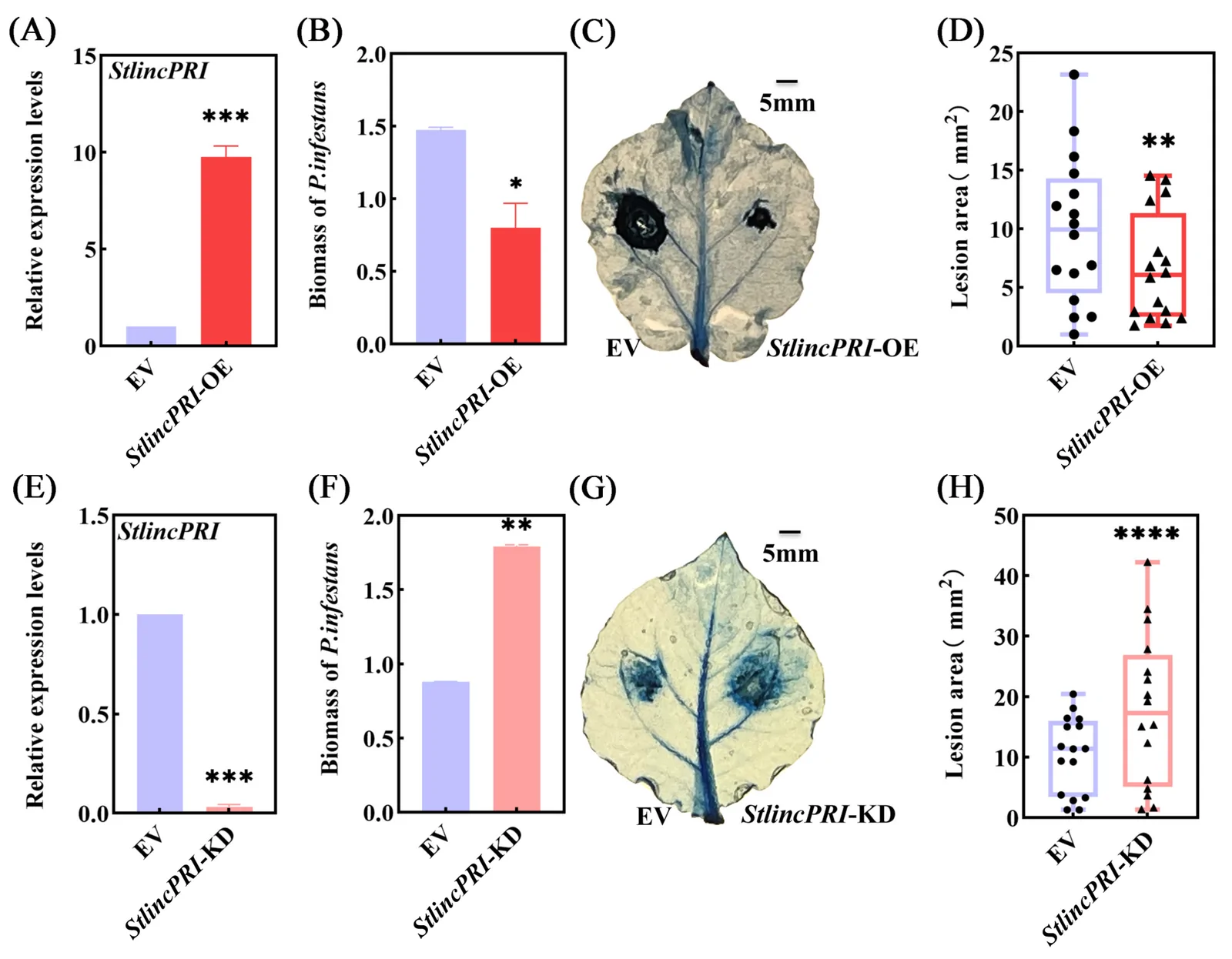
Effects of *StlincPRI* overexpression and silencing on potato resistance to *P. infestans*. (**A**,**E**) Relative expression levels of *StlincPRI* following transient transformation. (**B**,**F**) Relative *P. infestans* biomass estimated by qPCR targeting the *P. infestans*-specific element *PiO8*. (**C**,**G**) Representative trypan blue-stained detached leaves. Scale bars = 5 mm. (**D**,**H**) Quantification of lesion area. Boxes represent the 25th–75th percentiles, centre lines indicate the medians, and whiskers extend to the minimum and maximum values. Each point represents one leaf. Fifteen leaves from five independent plants were evaluated per treatment in each experiment; leaves from the same plant were subsamples. EV, empty vector control; *StlincPRI*-OE, *StlincPRI* overexpression; *StlincPRI*-KD, *StlincPRI* silencing. Data in (**A**,**B**,**E**,**F**) are presented as mean ± SD (*n* = 5). Statistical comparisons were performed between each treatment and its corresponding EV control using Student’s *t*-test. * *p* < 0.05; ** *p* < 0.01; *** *p* < 0.001; **** *p* < 0.0001.

Next, the function of *StlincPRI* in potato against *P. was investigated infestans* using detached leaves. At 48 h after transient transformation, *StlincPRI*-OE potato leaves showed 9-fold higher *StlincPRI* RNA level than control leaves (Figure 4A), while *StlincPRI* RNA-interference knockdown (*StlincPRI*-KD) leaves expressed only 0.8-fold *StlincPRI* RNA level compared to control (Figure 4E). After inoculation with *P. infestans*, *StlincPRI*-OE leaves showed decreased pathogen biomass (~0.5×) and lesion area (~0.5×) compared with control (Figure 4B–D). In contrast, *StlincPRI*-KD leaves showed increased pathogen biomass (~2.0×) and lesion area (~1.5×) compared with control (Figure 4F–H). These results demonstrate that *StlincPRI* enhances potato resistance to *P. infestans*.

**Figure 5:**
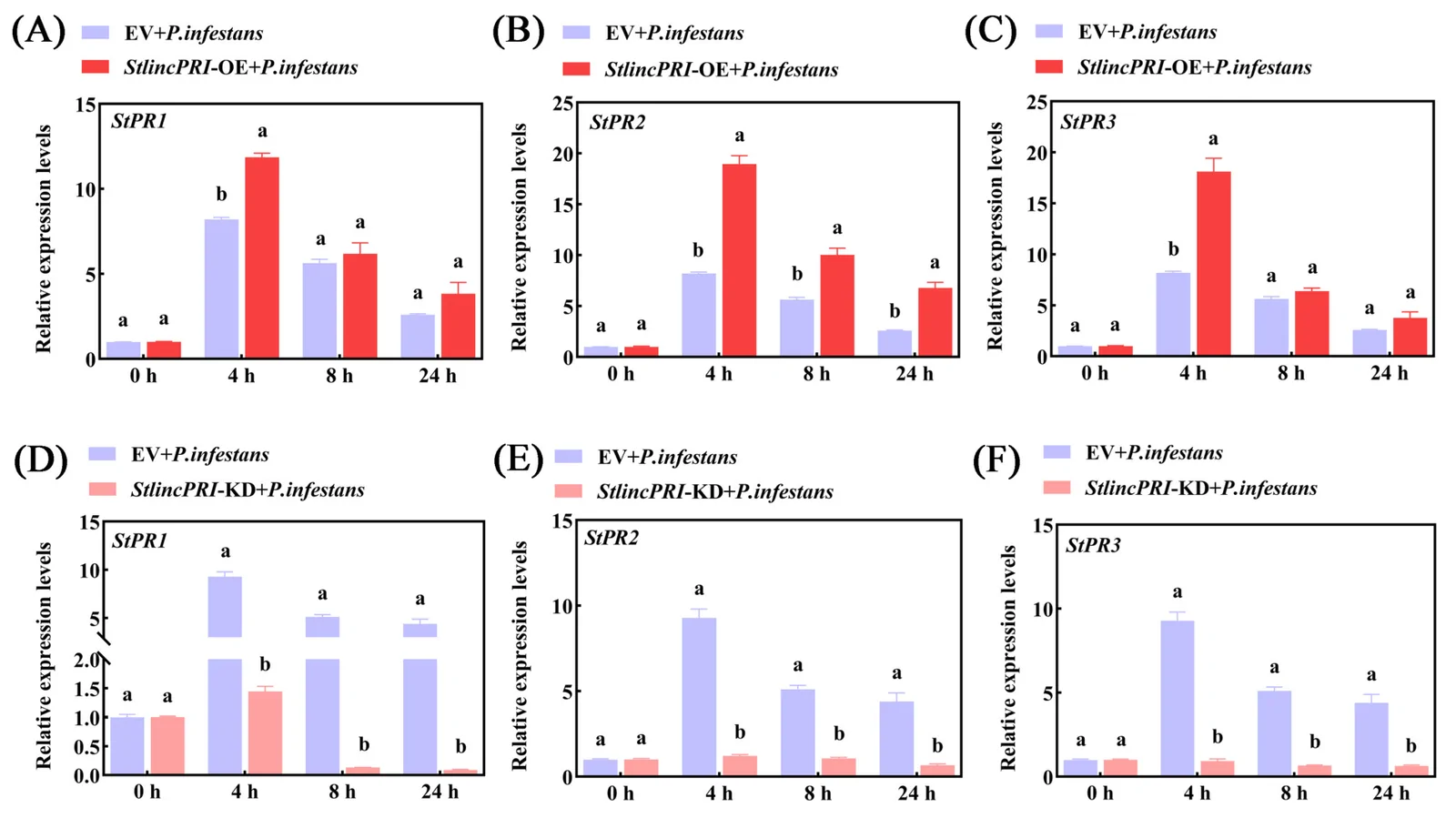
*PR* gene expression following *P. infestans* inoculation. (**A**–**C**) Relative expression of *StPR1*, *StPR2*, and *StPR3* in *StlincPRI*-OE leaves and the corresponding EV controls. (**D**–**F**) Relative expression of these genes in *StlincPRI*-KD leaves and the corresponding EV controls. Data are means ± SD (*n* = 3) from three independent biological replicates. Each panel was analyzed using ordinary two-way ANOVA with treatment, time, and their interaction, followed by Šídák-adjusted treatment–control comparisons across the four time points. Within each time point in a given panel, different letters indicate a significant treatment–control difference (*p* < 0.05); shared letters indicate no statistically significant difference.

To assess effects of *StlincPRI* on the immune response in potato, *PR* gene expression was measured. The results showed that potato *PR1*, *PR2*, and *PR3* were significantly upregulated (by approximately 2-fold at 4 hpi) in *StlincPRI*-OE leaves compared with the control (Figure 5A–C). In contrast, potato *PR1*, *PR2*, and *PR3* remained essentially un-induced by *P. infestans* infection and markedly lower (by up to 9-fold at 4 hpi) in *StlincPRI*-KD leaves compared to control (Figure 5D–F). These results indicate that *StlincPRI* is required for induction of *PR1*, *PR2*, and *PR3* expression in potato upon *P. infestans* infection.

### 2.5. StlincPRI Boosts ROS Burst in Potato Following P. infestans Infection

**Figure 6:**
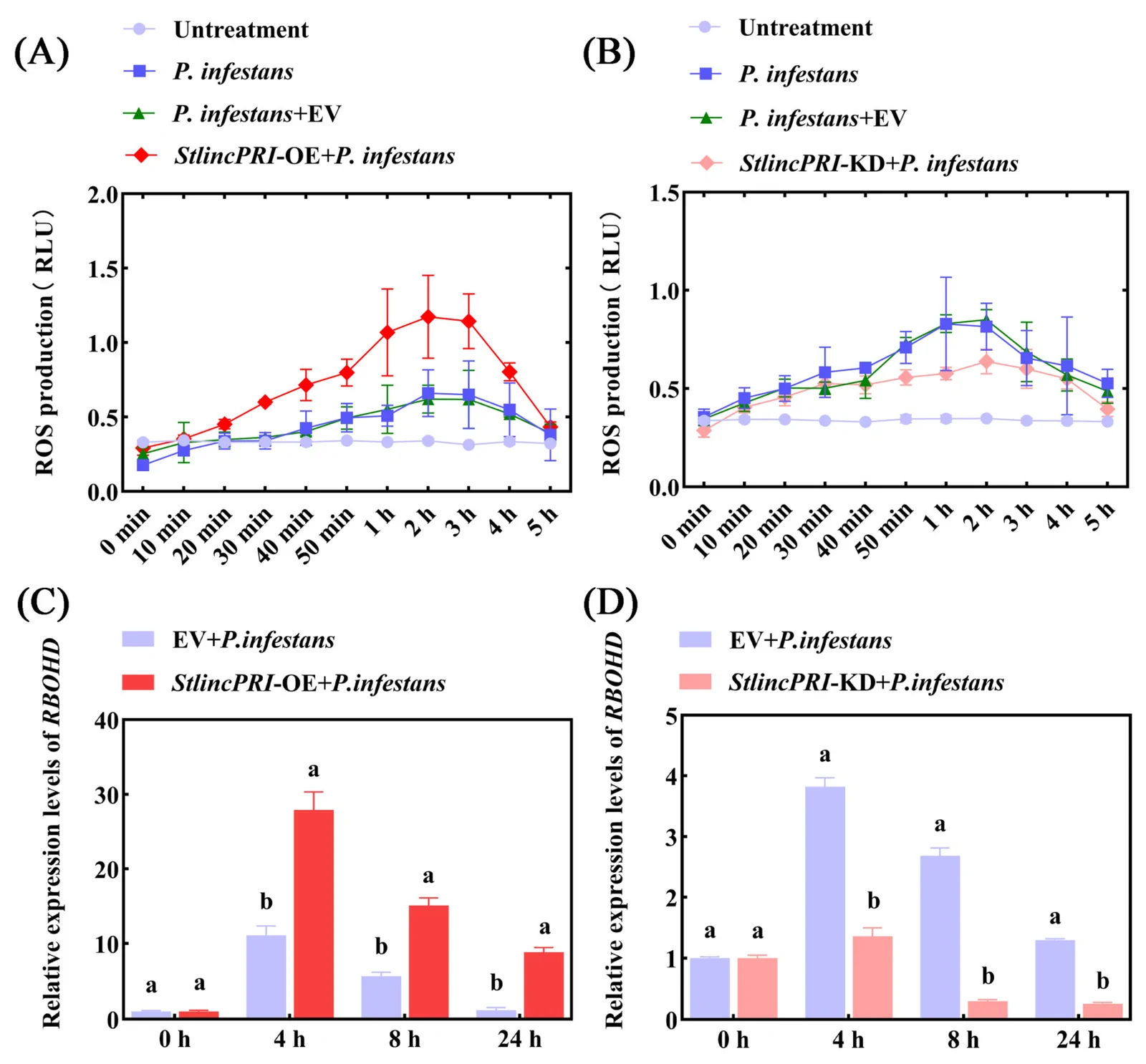
ROS bursts and *StRBOHD* expression levels following *P. infestans* infection. (**A**) ROS dynamics in *StlincPRI*-OE potato leaves. (**B**) ROS dynamics in *StlincPRI*-KD potato leaves. (**C**) *StRBOHD* expression in *StlincPRI*-OE potato leaves. (**D**) *StRBOHD* expression in *StlincPRI*-KD potato leaves. Data are presented as mean ± SD (*n* = 3) from three independent biological replicates. For *StRBOHD* expression (**C**,**D**), the overexpression and knockdown datasets were analyzed separately using ordinary two-way ANOVA with treatment, time, and their interaction. The four treatment–control comparisons at 0, 4, 8, and 24 hpi were jointly adjusted using Šídák’s method within each dataset. Different letters indicate significant differences between the treatment and EV control at the same time point (*p* < 0.05); shared letters indicate no statistically significant difference.

ROS can act as signaling molecules to transmit defense signals during plant immune responses. A ROS burst was detected at 2 hpi; moreover, *StlincPRI*-OE plants displayed a ROS burst 2.3-fold higher than control upon *P. infestans* infection (Figure 6A). In contrast, *StlincPRI*-KD plants displayed a ROS burst significantly lower (0.25×) than the control (Figure 6B).

RBOHs are a class of NADPH oxidases that catalyze the reduction of oxygen to superoxide, among which RBOHD is primarily responsible for ROS production during plant immune responses. *StRBOHD* RNA expression levels were examined in potato leaves at 0, 4, 8, and 24 hpi with *P. infestans*. In *StlincPRI*-OE plants, *StRBOHD* RNA levels were significantly (by 2~3-fold) higher than those in the control at the sampled post-inoculation time points (Figure 6C), whereas in *StlincPRI*-KD plants, *StRBOHD* RNA levels were consistently significantly lower (by 3~7-fold) compared with the control (Figure 6D). These results show that *StlincPRI* enhances the early ROS burst and is associated with increased *StRBOHD* transcript abundance following inoculation.

### 2.6. StlincPRI Elevates the ROS Scavenging Capacity in Potato Following P. infestans Infection

**Figure 7:**
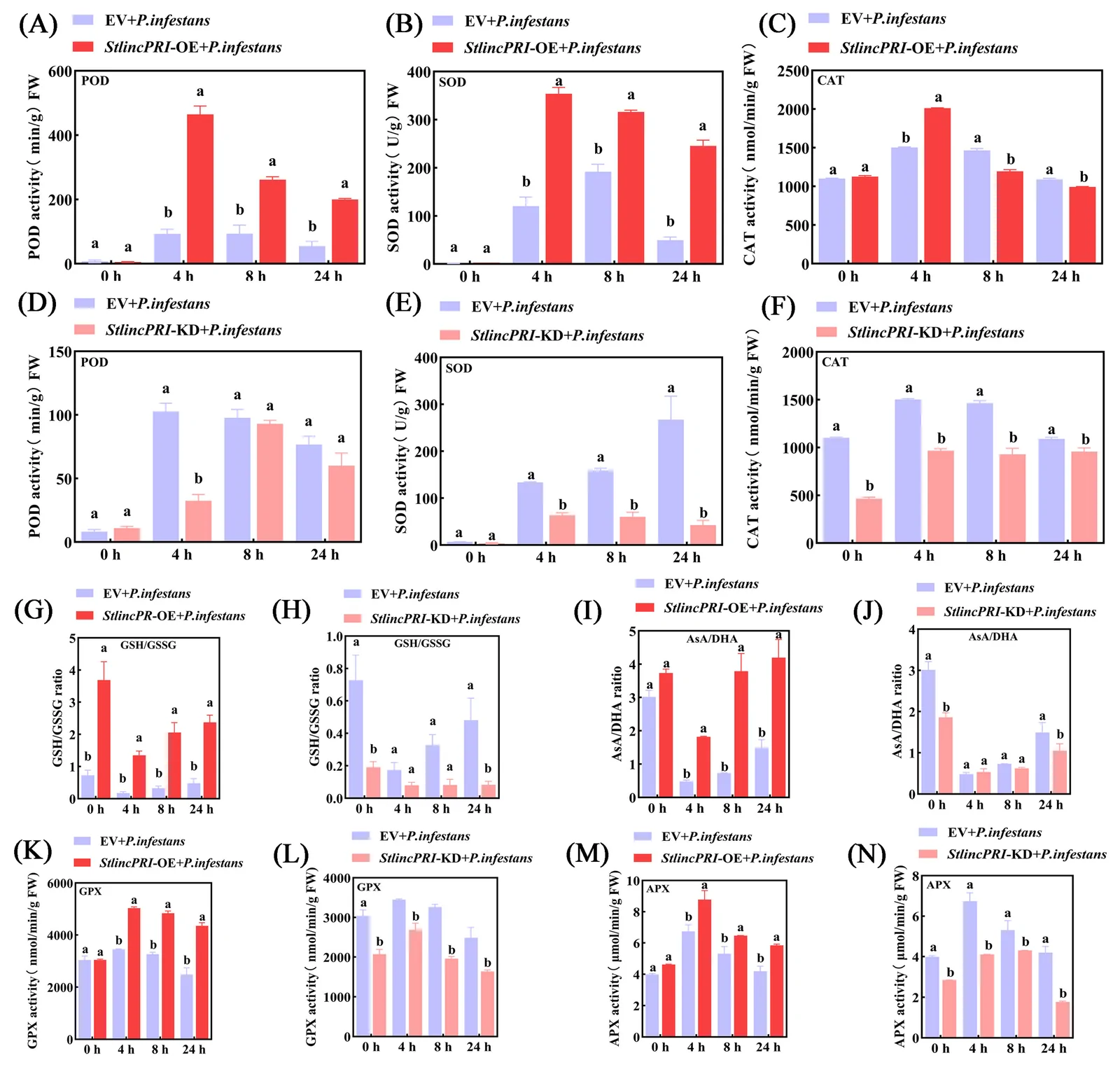
Antioxidant enzyme activities and redox ratios following *P. infestans* infection. (**A**–**C**) POD, SOD, and CAT activities in *StlincPRI*-OE leaves. (**D**–**F**) POD, SOD, and CAT activities in *StlincPRI*-KD leaves. (**G**,**H**) GSH/GSSG ratios. (**I**,**J**) AsA/DHA ratios. (**K**,**L**) GPX activities. (**M**,**N**) APX activities. (**G**,**I**,**K**,**M**) show overexpression experiments, whereas (**H**,**J**,**L**,**N**) show knockdown experiments, each with the corresponding empty-vector (EV) control. Data are presented as mean ± SD (*n* = 3) from three independent biological replicates. Each measured variable was analyzed separately for the overexpression and knockdown experiments using ordinary two-way ANOVA, including treatment, time, and their interaction. Within each dataset, the four treatment–control comparisons across time points were jointly adjusted using Šídák’s method. Different letters indicate significant treatment–control differences at the same time point (*p* < 0.05); shared letters indicate no statistically significant difference.

In *StlincPRI*-OE leaves, peroxidase (POD) and superoxide dismutase (SOD) activities were significantly higher than the control at 4, 8, and 24 hpi (Figure 7A,B). Catalase (CAT) activity was significantly higher at 4 hpi but lower at 8 and 24 hpi (Figure 7C). In *StlincPRI*-KD leaves, SOD and CAT activities were significantly lower at all three post-inoculation time points, whereas POD activity was significantly lower only at 4 hpi (Figure 7D–F).

*StlincPRI*-OE leaves exhibited significantly higher ratios of reduced to oxidized glutathione (GSH/GSSG) and ascorbate to dehydroascorbate (AsA/DHA) at 4, 8, and 24 hpi (Figure 7G,I). Glutathione peroxidase (GPX) and ascorbate peroxidase (APX) activities were also significantly higher at these time points (Figure 7K,M). In *StlincPRI*-KD leaves, GPX and APX activities were significantly lower at these time points (Figure 7L,N). However, among the post-inoculation measurements, GSH/GSSG and AsA/DHA ratios were significantly lower only at 24 hpi (Figure 7H,J). These findings suggest that *StlincPRI* influences glutathione and ascorbate redox balance and the activities of GPX and APX.

### 2.7. StlincPRI Reduces Oxidative Damage in Potato Following P. infestans Infection

**Figure 8:**
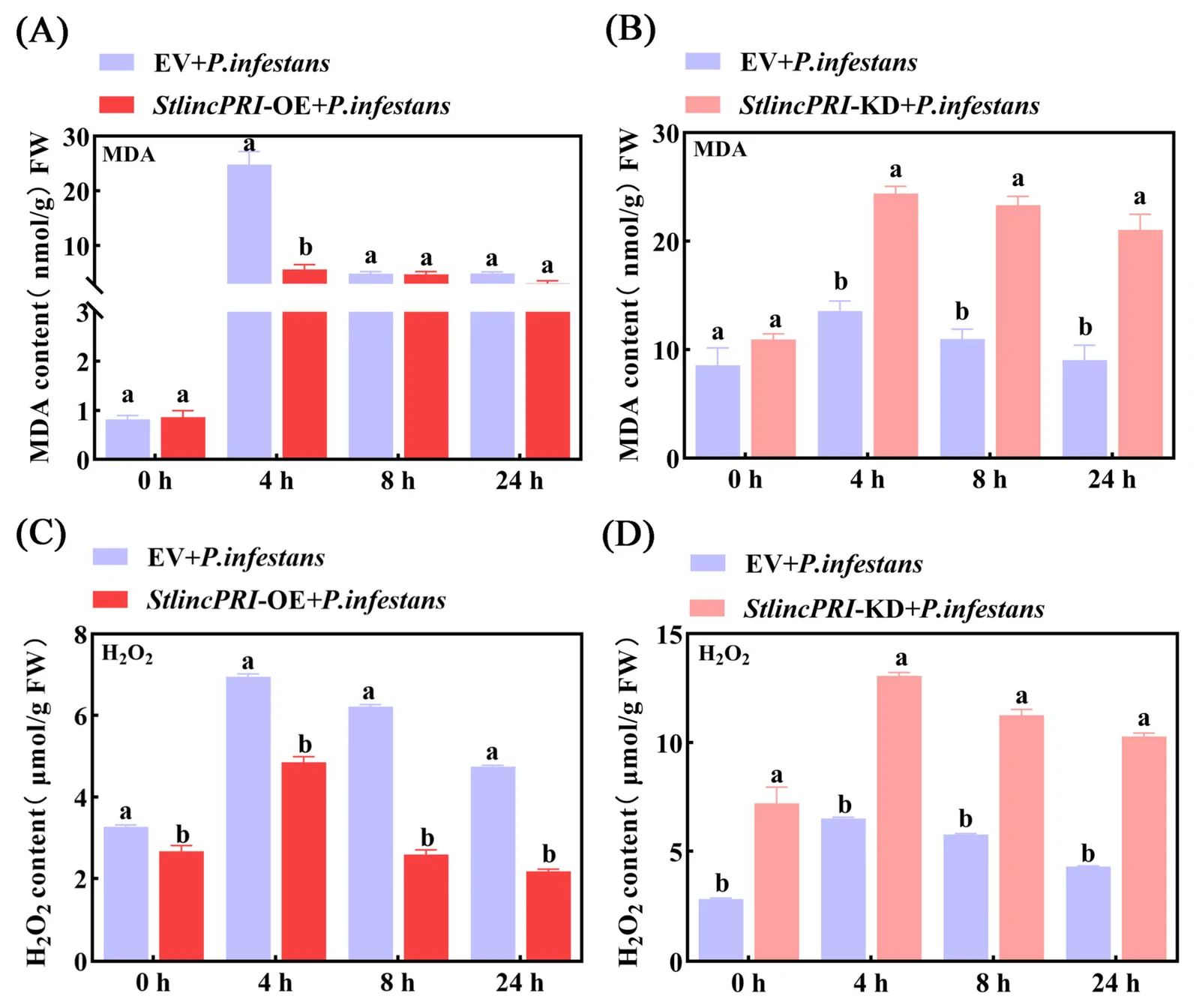
MDA and H_2_O_2_ contents in potato leaves following *P. infestans* infection. (**A**,**B**) MDA contents in *StlincPRI*-OE and *StlincPRI*-KD leaves, respectively. (**C**,**D**) H_2_O_2_ contents in *StlincPRI*-OE and *StlincPRI*-KD leaves, respectively. Each experiment included its corresponding empty-vector (EV) control. Data are presented as mean ± SD (*n* = 3) from three independent biological replicates. Each measured variable was analyzed separately for the overexpression and knockdown experiments using ordinary two-way ANOVA, including treatment, time, and their interaction. Within each dataset, the four treatment–control comparisons across time points were jointly adjusted using Šídák’s method. Different letters indicate significant treatment–control differences at the same time point (*p* < 0.05); shared letters indicate no statistically significant difference.

To assess the effects of *StlincPRI* on ROS accumulation and oxidative damage, we measured malondialdehyde (MDA) and H_2_O_2_ contents in potato leaves. In *StlincPRI*-OE leaves, MDA content was significantly lower than the control at 4 hpi, whereas differences at 8 and 24 hpi were not statistically significant (Figure 8A). In *StlincPRI*-KD leaves, MDA content was significantly higher than the control at 4, 8, and 24 hpi (Figure 8B). H_2_O_2_ content was significantly lower in *StlincPRI*-OE leaves and higher in *StlincPRI*-KD leaves at all sampled time points, including before inoculation (Figure 8C,D). Together, these results show that *StlincPRI* enhances the early ROS burst at approximately 2 hpi, whereas increased antioxidant capacity and reduced H_2_O_2_ and MDA accumulation are observed at 4 hpi.

### 2.8. Proposed Model of StlincPRI-Mediated Resistance to P. infestans

**Figure 9:**
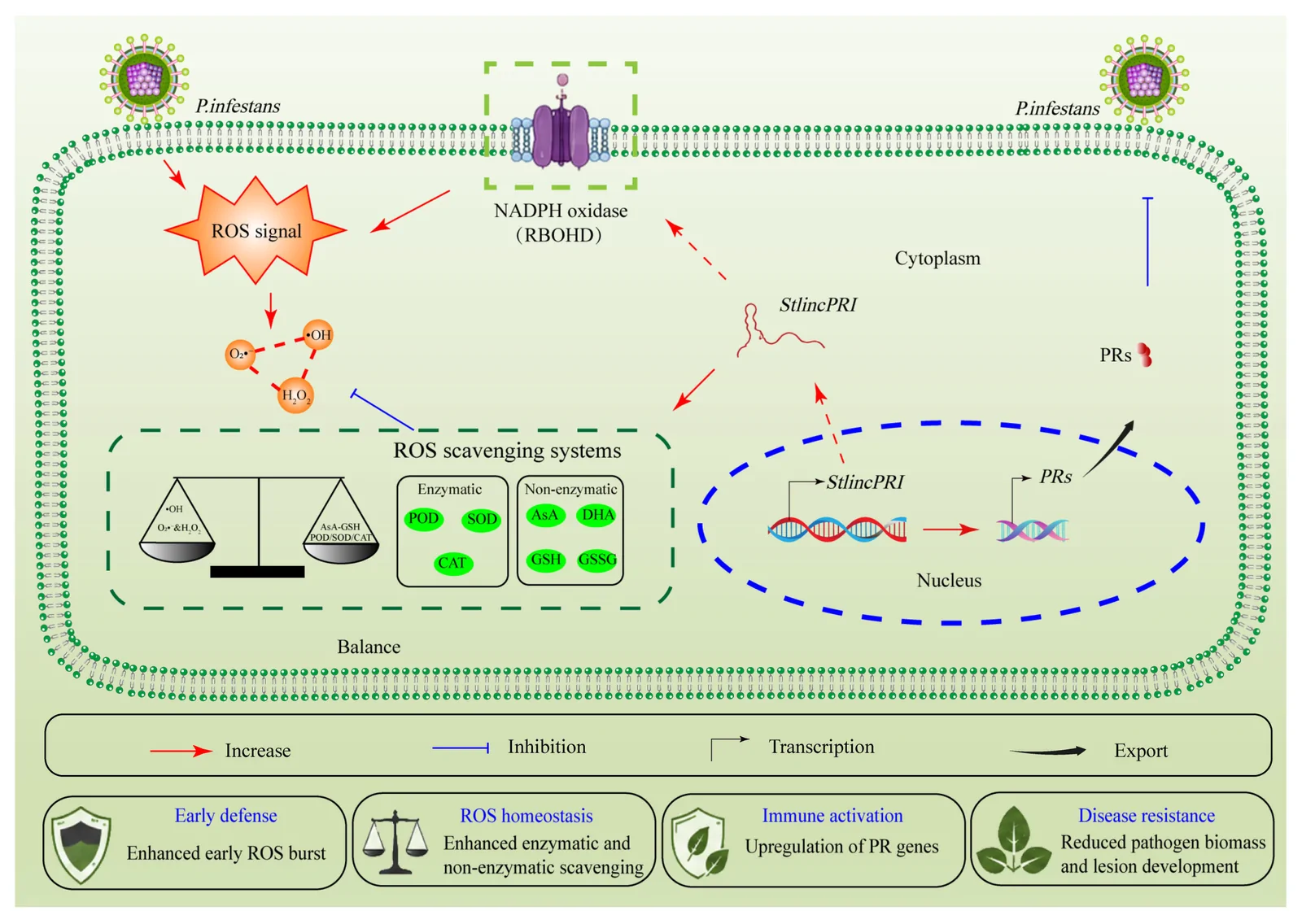
Model of regulatory mechanism of *StlincPRI* in potato resistance to *P. infestans.* Solid arrows indicate experimentally supported effects and do not necessarily imply direct regulation. Dashed arrows indicate proposed relationships that remain to be established.

Through a comprehensive analysis of the experimental results, we constructed a mechanistic model illustrating how *StlincPRI* enhances potato resistance via the regulation of ROS production and scavenging. Upon infection by *P. infestans*, the expression of *StlincPRI* is significantly upregulated. *StlincPRI* promotes *StRBOHD* expression and enhances the early ROS burst, although a causal link between these effects remains to be established. In addition, *StlincPRI* enhances the expression of PR genes. At the same time, to prevent oxidative damage caused by excessive ROS accumulation, *StlincPRI* activates the antioxidant enzyme system and the AsA–GSH cycle, facilitating the removal of excess ROS, maintaining intracellular redox homeostasis, and reducing membrane lipid peroxidation damage. Ultimately, these processes contribute to enhanced resistance against *P. infestans* (Figure 9).

## 3. Discussion

Accumulating evidence indicates that lncRNAs, once regarded as transcriptional noise, are now recognized as important regulators of gene expression. lncRNAs can negatively regulate plant resistance to pathogens. In cotton seedlings, silencing *GhlncNAT-ANX2* and *GhlncNAT-RLP7* enhances resistance to *Verticillium dahliae* and *Botrytis cinerea* [13]. In *Arabidopsis thaliana*, lncRNA *ELENA11* negatively regulates *PRLIP5* expression and suppresses callose deposition. Overexpression of *ELENA11* increased plant susceptibility to *P. syringae* pv. *tomato*, whereas disruption of *ELENA11* enhanced disease resistance [14]. Nevertheless, lncRNAs may also contribute positively to plant immune responses. For instance, in tomato, lncRNA33732, lncRNA39026, lncRNA08489, lncRNA20718 and lncRNA47980 have been reported to participate in resistance to *P. infestans* [15,16,17,18,19]. In rice, *ALEX1* contributes to immunity against *Xanthomonas oryzae* pv. *oryzae* [20], and *OslncBR3* has been implicated in resistance to *Magnaporthe oryzae* [21]. In potato, lncRNA13558 promotes resistance to *P. infestans* [12]. Here, *StlincPRI* was identified as an intergenic lncRNA that enhances potato resistance to *P. infestans*. Functional analyses using gain- and loss-of-function methods demonstrated that *StlincPRI* suppresses lesion expansion and restricts *P. infestans* growth. Additionally, *StlincPRI* upregulates expression levels of *PR1*, *PR2*, and *PR3*. *PR* genes are widely regarded as classical markers of plant innate immunity. They are rapidly upregulated during the stages of pathogen infection, and their expression is often closely associated with phytohormones, like salicylic acid and jasmonic acid (JA), as well as ROS bursts [22,23]. SA-dependent pathways activate *PR* genes, including *PR1*, *PR2*, and *PR5*, to fight against biotrophic and hemibiotrophic pathogens [24,25]. By contrast, JA/ethylene (ET)-mediated pathways boost *PR3*, *PR4*, and *PR12* expression to defend against invasion by necrotrophic pathogens [26]. In this study, it was also assessed expression levels of *PR4*, *PR5*, and *PR17*, but the results differed from those for *PR1*, *PR2*, and *PR3*. At 4 hpi and 8 hpi with *P. infestans*, *PR4*, *PR5*, and *PR17* expression levels in *StlincPRI* overexpression leaves were significantly lower than control (Figure S5). Thus, *PR4*, *PR5*, and *PR17* may not be involved in *StlincPRI*-mediated resistance to *P. infestans*.

It was found that *StlincPRI* triggered a rapid burst of ROS and peaked at 2 hpi with *P. infestans*. ROS function both as signaling molecules to activate downstream defense genes and as direct antimicrobial agents [27]. Accumulating evidence indicates that ROS bursts are closely associated with disease resistance. In the rice *Magnaporthe oryzae* interaction, localized ROS accumulation at infection sites suppresses expression of effector BAS4 and restricts invasive hyphal growth, limiting pathogen colonization and enhancing host resistance [28]. Similarly, in potato, StlncRNA13558 regulates expression of pathogenesis-related protein StPRL, leading to increased ROS accumulation and activation of the SA signaling pathway, ultimately enhancing disease resistance [12]. In tomato, SllncRNA33732 upregulates transcription factor WRKY1, which in turn activates NADPH oxidase gene *SlRBOH*, thereby promoting ROS accumulation and enhancing plant immunity [15]. RBOHD represents a major source of ROS during plant immune responses. They belong to a class of NADPH oxidases that catalyze reduction of O_2_ to superoxide anions, which are subsequently dismutated into H_2_O_2_ [29]. Increasing evidence suggests that RBOHD positively regulates plant immunity through ROS-mediated defense responses. In *Arabidopsis*, BIK1-mediated phosphorylation of RBOHD plays a crucial role in ROS burst and antibacterial immunity [30]. Accumulation of RBOHD in *Arabidopsis pbl13* and *pire* mutants was associated with enhanced resistance to bacterial pathogens [31]. Following *P. infestans* inoculation, *StlincPRI* overexpression increased *StRBOHD* transcript abundance. The ROS chemiluminescence response peaked at approximately 2 hpi, whereas *StRBOHD* transcript abundance was highest at 4 hpi among the sampled time points. *StRBOHD* expression may already have been elevated at 2 hpi, potentially contributing to the early ROS response; however, expression was not measured at this time point, and its temporal relationship with the ROS burst remains unresolved.

ROS function as key defense-associated signals in plants, yet their excessive accumulation can impose oxidative damage on cellular components. Thus, effective immune responses require precise regulation of ROS production and removal, allowing ROS to participate in defense signaling while preventing oxidative toxicity. Maintenance of ROS homeostasis relies on an integrated antioxidant network that includes enzymatic scavengers, such as SOD, POD and CAT, as well as non-enzymatic systems, including the AsA–DHA and GSH–GSSG cycles [32,33]. When ROS are overproduced, AsA reduces cellular H_2_O_2_ to water while being oxidized to DHA. Subsequently, DHA is reduced back to AsA with the assistance of GSH, during which GSH is oxidized to GSSG. GSSG is then reduced back to GSH by glutathione reductase (GR), maintaining the ascorbate-glutathione cycle to continuously eliminate ROS and protect cells from oxidative damage. It has been reported that potato late blight resistance is closely associated with the dynamic balance between ROS production and antioxidant protection. The resistant cultivar Ovatio maintains this equilibrium through transient H_2_O_2_ bursts and efficient SOD/CAT/APX scavenging, whereas susceptible cultivar Bintje fails to coordinate this response [34]. *StlincPRI* overexpression increased several antioxidant enzyme activities and the GSH/GSSG and AsA/DHA ratios following infection, consistent with enhanced antioxidant capacity. However, CAT activity was higher than the control at 4 hpi but lower at 8 and 24 hpi, indicating distinct temporal responses among antioxidant components. Conversely, *StlincPRI* silencing reduced several antioxidant parameters. H_2_O_2_, as a representative ROS, and MDA, a lipid peroxidation product, are used to evaluate oxidative damage. Following inoculation, *StlincPRI*-OE leaves had lower H_2_O_2_ contents at 4, 8, and 24 hpi and lower MDA content at 4 hpi, whereas *StlincPRI*-KD leaves had higher contents of both at all three time points. These results further indicate that *StlincPRI* enhances the ROS-scavenging capacity of potato through both enzymatic and non-enzymatic antioxidant systems following *P. infestans* infection, which alleviates ROS-induced oxidative damage. Notably, this coordinated regulation of ROS production and scavenging supports the concept that an optimal balance between ROS generation and detoxification is essential for plant immunity, as previously proposed [35,36]. Similar regulatory patterns have also been observed in other plant–pathogen systems, such as OsmiR528-mediated ROS homeostasis in rice [37]. However, measurements beginning at 4 hpi do not establish the onset of antioxidant responses, and ROS production and scavenging may overlap.

Previous studies have shown that lincRNAs can modulate the expression of proximal protein-coding genes. Transcriptome analysis of potato infected with ‘*Ca.* L. solanacearum’ suggested that lincRNAs act in cis to regulate neighboring genes involved in defense responses [7]. RNA-seq analysis identified lincRNAs associated with *Ralstonia solanacearum* infection in tomato; these lincRNAs may contribute to disease resistance by cis-regulating neighboring genes involved in jasmonic acid, ethylene, and AGO-related pathways [9]. As an intergenic lncRNA, *StlincPRI* may regulate the expression of neighboring genes. To explore this possibility, we examined the transcriptional changes of the flanking genes Soltu.DM.12G024930.1 and Soltu.DM.12G024940.1 following *P. infestans* infection (Figure S6). The results showed that overexpression of *StlincPRI* led to a coordinated upregulation of both upstream and downstream genes, whereas silencing of *StlincPRI* had little effect on their expression. *StlincPRI* may influence the expression of neighboring genes, but whether this effect involves cis-regulation remains unclear.

## 4. Materials and Methods

### 4.1. Plant Cultivation and Treatment Conditions

Aseptic plantlets of the late blight-resistant potato cultivar Qingshu No. 9 and the susceptible cultivar Désirée were cultured on MS medium [38] for approximately 45 days. Both cultivars were maintained and provided by the College of Life Sciences, Inner Mongolia Agricultural University, Hohhot, China. *P. infestans* was propagated on oat agar medium at 20 °C under dark conditions. Mycelia were rinsed with sterile distilled water, and the released zoospores were harvested and diluted to 1 × 10^6^ spores mL^−1^. The zoospore suspension was uniformly applied to tissue-cultured plantlets of both potato cultivars. Leaf samples were harvested before inoculation (0 hpi) and at 4 hpi and 24 hpi, with three independent biological replicates collected for each cultivar at each time point. Total RNA was extracted and subjected to whole-transcriptome sequencing. *N. benthamiana* seeds were germinated on MS medium and subsequently transferred to nutrient-rich soil for further growth. Plants derived from tubers of the cultivar Wotu No. 5 were grown under controlled conditions at 22 °C ± 3 °C with a 16 h light/8 h dark photoperiod and used for transient expression assays in leaves.

### 4.2. RNA Extraction, Library Construction and Sequencing

Total RNA was extracted using TRIzol reagent (Invitrogen, Thermo Fisher Scientific, Waltham, MA, USA) according to the manufacturer’s instructions. RNA integrity and quality were assessed using agarose gel electrophoresis and an Agilent 2100 Bioanalyzer (Agilent Technologies, Santa Clara, CA, USA). For mRNA and lncRNA sequencing, ribosomal RNA was removed, and strand-specific libraries were constructed. For small RNA sequencing, libraries were prepared using the TruSeq Small RNA Sample Prep Kit (Illumina, San Diego, CA, USA) according to the manufacturer’s instructions. All libraries were sequenced on an Illumina platform to generate paired-end reads (for mRNA/lncRNA) and single-end reads (for small RNA).

### 4.3. Transcriptome Data Processing and Differential Expression Analysis

Sequencing reads were processed using Cutadapt v1.10, and read quality was assessed using FastQC v0.10.1. Filtered reads were aligned to the *Solanum tuberosum* Group Phureja DM 1-3 516 R44 reference genome v6.1 using HISAT2 v2.0.4. Transcripts were assembled and merged using StringTie v1.3.0. Candidate long non-coding RNAs (lncRNAs) were identified by excluding known messenger RNAs (mRNAs) and transcripts predicted to have protein-coding potential. Retained transcripts were required to be at least 200 nt long, have read coverage of at least 3, and contain at least one exon. Coding potential was assessed using CPC v0.9-r2 and CNCI v2.0. Transcript class codes and exon structures were obtained from the merged annotation in Gene Transfer Format (GTF). Transcripts assigned class code “u” were classified as intergenic. Expression levels of mRNAs and lncRNAs were quantified as fragments per kilobase of transcript per million mapped fragments (FPKM), and normalized read counts were obtained for microRNAs (miRNAs). Differential expression analysis was performed using DESeq2 v1.46.0. Genes with |log_2_(fold change)| > 1 and *p* < 0.05 were considered differentially expressed. The union of differentially expressed genes from the four within-cultivar comparisons was analyzed using custom R v4.6.0 scripts to cluster transcripts by their temporal expression patterns. The analysis covered three sampling time points in each cultivar, comprising six conditions: D0 hpi, D4 hpi, D24 hpi, Q0 hpi, Q4 hpi, and Q24 hpi. Expression profiles and cluster assignments are provided in Table S1.

Candidate prioritization focused on cluster 2 transcripts represented in the parameter1 column of the supplied correlation table. Entries with complete six-condition expression profiles and cluster membership values were retained. Duplicate entries were collapsed after confirming identical expression profiles and membership values. Each supplied expression profile had a mean of zero and a sample standard deviation of one across the six conditions and was used without further standardization. To identify transcriptsshowing a peak specifically at 4 hpi in Qingshu No. 9, an idealized template was defined as *v* = (0, 0, 0, 0, 1, 0), corresponding to D0 hpi, D4 hpi, D24 hpi, Q0 hpi, Q4 hpi and Q24 hpi, respectively. For each transcript, the template-similarity score was calculated as the Pearson correlation between its standardized expression vector and the template. Transcripts with similarity scores > 0.99 were retained and ranked in descending order of their membership in cluster 2. The transcript with the highest membership among the retained candidates was prioritized. Template similarity was treated as a descriptive measure of expression-pattern concordance rather than subjected to significance testing. Sensitivity to the similarity threshold was assessed using cutoffs of 0.970, 0.980, 0.985, 0.990, 0.995, and 0.996. Candidate-prioritization results and threshold-sensitivity analyses are provided in Tables S4 and S5, respectively. These calculations and visualizations were performed using R v4.6.0. For transcript-specific characterization, the genomic context of *StlincPRI* was examined against the DM v6.1 high-confidence gene annotation. Its 456-nt sequence was verified against the assembled-transcript and candidate-lncRNA FASTA files. Additional coding-potential assessment used the feature-extraction routines, scaling parameters, and pretrained model distributed with CPC2 standalone v1.0.1. The analysis was implemented through a local Python v3.9.1 adapter using Biopython v1.88 and LIBSVM v3.37.0. The sequence was analyzed in its transcript orientation, and the coding probability and classification were recorded.

### 4.4. RT-qPCR Analysis

RT-qPCR was performed to evaluate the sequencing-based transcriptome data and verify their reliability. Total RNA was extracted from potato leaves using the RNAiso Plus reagent kit (TaKaRa, Dalian, China https://www.takarabio.com/, accessed on 17 October 2026), and genomic DNA was extracted using the Plant Genomic DNA Extraction Kit (TIANGEN Biotech, Beijing, China, http://www.tiangen.com/en/, accessed on 17 October 2026). First-strand cDNA was synthesized from RNA with PrimeScript™ RT Master Mix (TaKaRa, Dalian, China; cat. no.6210A), and RT-qPCR was carried out using SYBR Mix according to the manufacturer’s instructions (Vazyme, Nanjing, China; cat. no. Q411). Reactions were run on a LightCycler 480 system (Roche Diagnostics, Rotkreuz, Switzerland). Potato *EF1α* served as the internal reference to normalize lncRNA expression. The reference gene and primer information used for RT-qPCR are provided in Table S2. Relative expression levels were calculated using the 2^−ΔΔCt^ method, and results were based on three independent biological replicates.

### 4.5. Construction of StlincPRI Overexpression and Silencing Vectors

The *StlincPRI* sequence was synthesized by Beijing Tsingke Biological Co., Ltd. (Beijing, China), and cloned into the pUC57 cloning vector to generate the pUC57-StlincPRI cloning plasmid. All restriction enzymes used in this study were purchased from TaKaRa (Dalian, China). The target fragment was then excised by double digestion with KpnI and SacI and subcloned into the pRI101AN binary vector to construct the *StlincPRI* overexpression plasmid. For the silencing construct, a *StlincPRI*-specific fragment (355 bp) was PCR amplified with EcoRI and BamHI restriction sites appended to the 5′ and 3′ ends, respectively, and was co-ligated with a linker, which is a 500 bp fragment from the Gus gene containing an EcoRI site, into the pENTR D vector L16 to obtain the silencing framework plasmid with hairpin structure. Then the hairpin structure “StlincPRI-Gus-StlincPRI” fragment was enzymatically cleaved and inserted into the pRI101AN binary vector to generate the *StlincPRI* silencing construct, designated *StlincPRI*-KD. Primer sequences used for cloning are listed in Table S2. The vector backbone is shown in Figure S4.

### 4.6. Analysis of Potato and Tobacco Resistance Against P. infestans

*Agrobacterium* strains harboring the recombinant pRI101AN plasmids were resuspended in infiltration buffer (1 mL 1 M MES-KOH, pH 8.2; 1 mL 1 M MgCl_2_; 100 μL 100 mM acetosyringone). Leaves of the potato cultivar Wotu No. 5 at a uniform mid-vegetative stage were selected for *Agrobacterium* infiltration. The abaxial surface was gently abraded with fine sandpaper to create micro-wounds, and 5 mm filter-paper disks were placed on the wounded sites. Subsequently, 100 μL of *Agrobacterium* suspension carrying pRI101-StlincPRI or the silencing construct pRI101-AN-StlincPRI-KD was applied to each disk. Leaves infiltrated with *Agrobacterium* containing the empty vector served as controls. A zoospore suspension of *P. infestans* (1 × 10^6^ zoospores mL^−1^) was inoculated onto the leaf surface at 20 μL per site. For lesion area quantification, 15 detached leaves from five independent plants were evaluated per treatment in each experiment. The plants constituted the biological replicates, and leaves collected from the same plant were subsamples.

Transient expression assays were performed by infiltrating the prepared *Agrobacterium* suspensions into both leaf surfaces of *N. benthamiana* using a needleless syringe. For lesion area quantification, six detached leaves from three independent plants were evaluated per treatment. The plants constituted the biological replicates, and leaves collected from the same plant were subsamples. Lesion development was recorded, and lesion areas were quantified at 7 days post-inoculation. Trypan blue staining was performed on potato and tobacco leaves to detect tissue necrosis. MES, MgCl_2_, acetosyringone, and trypan blue were purchased from TransGen Biotech (Beijing, China).

### 4.7. Detection of ROS Burst and Scavenging

ROS were measured using a luminol-based chemiluminescence assay with minor modifications [39]. Leaves were first challenged with a *P. infestans* zoospore suspension at a concentration of 1 × 10^6^ zoospores mL^−1^, with 20 μL applied to each inoculation site. Leaf discs of 5 mm in diameter were then excised using a cork borer and immediately immersed in a freshly prepared luminol–horseradish peroxidase (HRP) reaction solution. The reaction solution was prepared in 10 mM phosphate buffer and contained 50 μM luminol and 10 μg mL^−1^ HRP. Luminol and HRP were purchased from Sigma-Aldrich (St. Louis, MO, USA). The reaction mixtures were subsequently transferred to 96-well plates, and chemiluminescence signals were recorded at 30 s intervals for at least 5 h to monitor the dynamics of the ROS burst.

Activities of POD, SOD, CAT, GPX and APX, as well as the contents of AsA, DHA, GSH, GSSG, H_2_O_2_ and MDA, were determined using commercial assay kits (Suzhou Grace Biotechnology Co., Ltd., Suzhou, China) according to the manufacturer’s instructions. ROS chemiluminescence and antioxidant enzyme activities were measured using a Synergy H4 multimode microplate reader (BioTek Instruments, Winooski, VT, USA).

### 4.8. Data Analysis

Statistical analyses and image processing were performed using GraphPad Prism 10.0.0 and ImageJ v1.54f, respectively. Lesion areas were quantified using ImageJ. Images were first calibrated using the 5 mm scale bar, after which lesion boundaries were manually delineated and the corresponding areas were calculated and expressed in mm^2^. Unless otherwise indicated, data are expressed as mean ± SD. Treatment–control comparisons at a single time point in the disease-resistance assays were assessed using Student’s *t*-tests. Time-course measurements of *PR* and *StRBOHD* expression, antioxidant-related parameters, and H_2_O_2_ and MDA contents were analyzed using ordinary two-way ANOVA, including treatment, time, and their interaction. Each response variable was analyzed separately for the overexpression and knockdown experiments. Treatment–control comparisons at individual time points were adjusted using Šídák’s method, with all sampled time points forming one comparison family for each response variable within each experiment. No adjustment was applied across response variables or between the overexpression and knockdown experiments. *p* values < 0.05 were considered statistically significant, using *p* values for multiple comparisons.

## 5. Conclusions

In this study, transcriptome profiling of the resistant potato cultivar Qingshu No. 9 and the susceptible cultivar Désirée, combined with gain- and loss-of-function analyses, identified *StlincPRI* as a *P. infestans*-responsive long intergenic non-coding RNA that positively regulates potato resistance to late blight. Overexpression of *StlincPRI* restricted *P. infestans* growth and lesion development, whereas silencing of *StlincPRI* resulted in increased pathogen biomass and disease symptoms. *StlincPRI* enhanced the early ROS burst and altered enzymatic and non-enzymatic antioxidant responses following infection. Together with the changes in tissue H_2_O_2_ and MDA contents, these findings support a role for *StlincPRI* in regulating ROS homeostasis during potato–*P. infestans* interaction. In parallel, *StlincPRI* promotes the expression of *PR1*, *PR2*, and *PR3*, further contributing to immune activation. These findings identify *StlincPRI* as a potential regulatory target for improving potato late blight resistance and provide a potential genetic resource for resistance breeding. Recommended next steps include determining the molecular mechanism by which *StlincPRI* regulates ROS homeostasis and PR gene expression, clarifying its potential cis-regulatory effects on neighboring genes, and validating its role in disease resistance using stable transgenic potato lines and under field conditions. Limitations of the present study include the reliance on transient overexpression and silencing assays, the lack of direct molecular evidence linking *StlincPRI* to its downstream targets, and the absence of validation using stable transgenic lines. Overall, these findings identify a previously uncharacterized lincRNA-mediated regulatory pathway involved in potato immunity through the coordination of ROS homeostasis and defense gene activation, providing a valuable foundation for the molecular breeding of late blight-resistant potato cultivars.

## Data Availability

Raw data of RNA-seq are available in the NCBI BioProject database (PRJNA1465629).

## Supplementary Materials

The following supporting information can be downloaded at https://www.mdpi.com/article/10.3390/plants15203191/s1. Figure S1: Volcano plots and hierarchical clustering heatmaps of differentially expressed genes in resistant and susceptible potato cultivars following *P. infestans* inoculation; Figure S2: Expression profiles of candidate transcripts matching the Q4h peak pattern; Figure S3: Genomic location and transcriptional orientation of *StlincPRI*; Figure S4: Vector backbones for *StlincPRI* overexpression and silencing; Figure S5: PR gene expression following *P. infestans* inoculation; Figure S6: Expression levels of genes flanking *StlincPRI*; Table S1: Expression profiles and cluster assignments of differentially expressed genes used for time-series clustering analysis; Table S2: Primers used in this study; Table S3: Structural characteristics and coding-potential assessment of MSTRG.40637.1 (*StlincPRI*); Table S4: Prioritization of cluster 2 transcripts based on expression profiles and cluster membership together with similarity to the Qingshu No. 9 4 h peak-expression template; Table S5: Sensitivity analysis of candidate prioritization across different template-similarity thresholds.
