# Supplementary material for: Long Non-Coding RNA StlincPRI Enhances Resistance to Phytophthora infestans by Regulating Reactive Oxygen Species Homeostasis
Source: Plants (Basel). 2026 Oct 18;15(20):3191. doi: 10.3390/plants15203191 (PMC13709009; doi:10.3390/plants15203191)
Supplement: Supplementary file 1 [file plants-15-03191-s001.zip › Supplementary-Figure S1-S6.pdf]

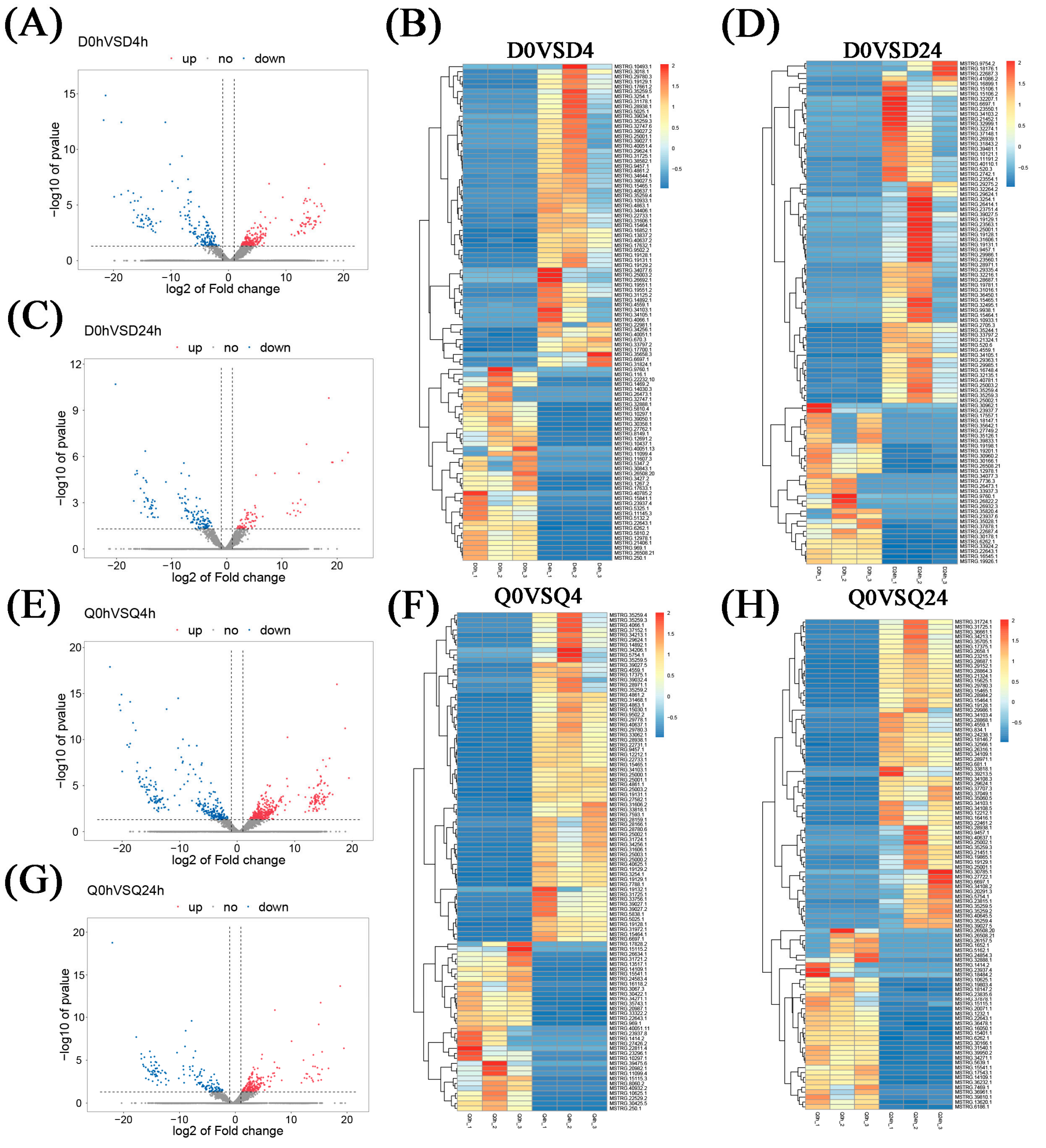

Figure S1. Volcano plots and hierarchical clustering heatmaps of differentially expressed genes in resistant and susceptible potato cultivars following *P. infestans* inoculation. (A, B) Volcano plot and hierarchical clustering heatmap of differentially expressed genes (DEGs) in the susceptible cultivar Désirée (D) at 4 hpi compared with 0 hpi (D4 hpi vs D0 hpi). (C, D) Volcano plot and hierarchical clustering heatmap of DEGs in Désirée at 24 hpi post-inoculation compared with 0 hpi (D24 hpi vs D0 hpi). (E, F) Volcano plot and hierarchical clustering heatmap of DEGs in the resistant cultivar Qingshu No. 9 (Q) at 4 hpi compared with 0 h (Q4 hpi vs Q0 hpi). (G, H) Volcano plot and hierarchical clustering heatmap of DEGs in Qingshu No. 9 at 24 hpi post-inoculation compared with 0 h (Q24 hpi vs Q0 hpi). DEGs were identified using thresholds of $|\log_2 FC| > 1$ and $P < 0.05$. In the volcano plots, red, blue and grey dots represent significantly upregulated, significantly downregulated and non-significantly changed genes, respectively. In the heatmaps, each row represents a DEG and each column represents an independent biological replicate; red and

blue indicate relatively high and low expression levels, respectively. Désirée and Qingshu No. 9 denote the susceptible cultivar Désirée and the resistant cultivar Qingshu No. 9, respectively, and 0, 4 and 24 hpi indicate the sampling times following *P. infestans* inoculation.

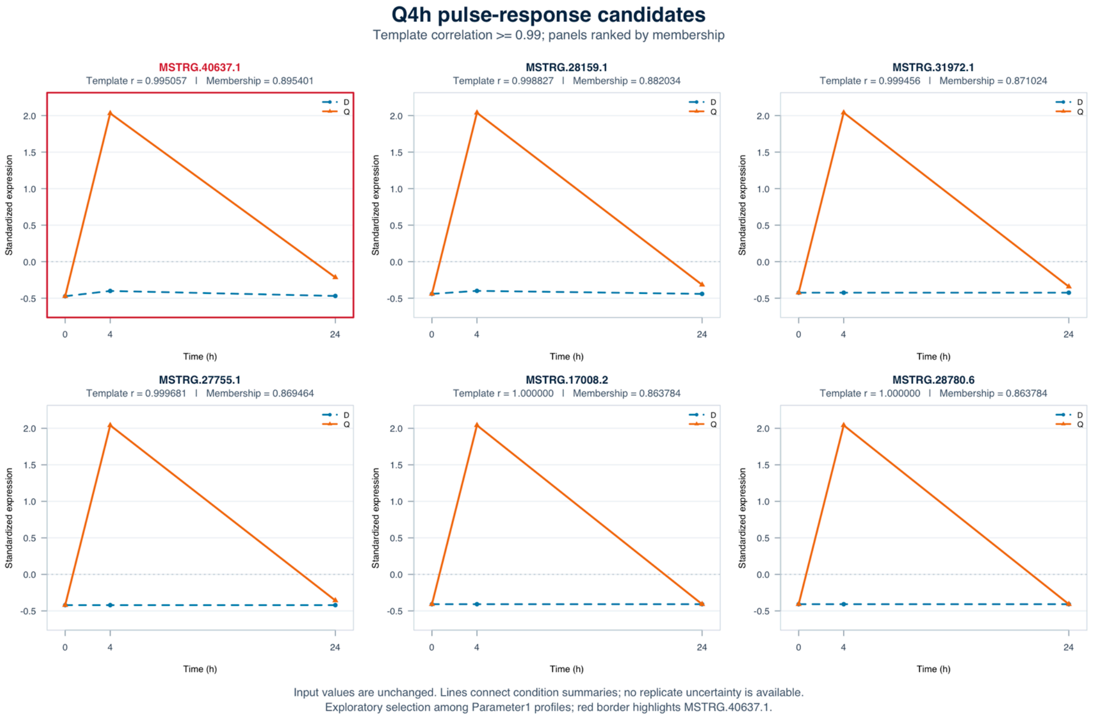

Figure S2. Expression profiles of candidate transcripts matching the Q4h peak pattern. Six transcripts with Pearson correlations of greater than 0.99 with the idealized template (0,0,0,0,1,0) are shown in descending order of cluster 2 membership. Blue dashed lines and orange solid lines represent conditions Désirée and Qinshu No.9, respectively. The red border highlights MSTRG.40637.1. Expression values are standardized within each transcript, and all panels share the same vertical scale. Template correlations and membership values are indicated above each panel. Lines connect the supplied condition-level values to illustrate expression patterns; no replicate-based uncertainty estimates are shown.

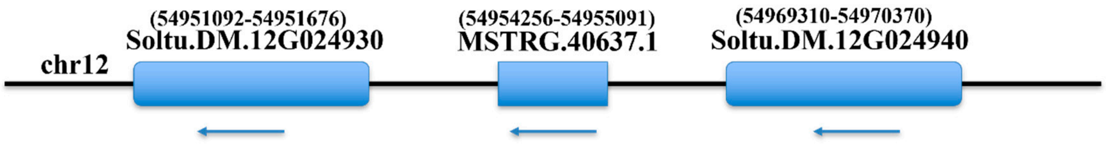

Figure S3. Genomic location and transcriptional orientation of *StlincPRI*. *StlincPRI* (MSTRG.40637.1) is located on the negative strand of chromosome 12 at positions 54,954,256–54,955,091 in the DM v6.1 reference. The assembled transcript contains two exons at positions 54,954,256–54,954,540 and 54,954,921–54,955,091, with a combined length of 456 nt. It lies between Soltu.DM.12G024930.1 (54,951,092–54,951,676) and Soltu.DM.12G024940.1 (54,969,310–54,970,370), which are also located on the negative strand. Coordinates are 1-based and inclusive. Arrows indicate transcriptional orientation.

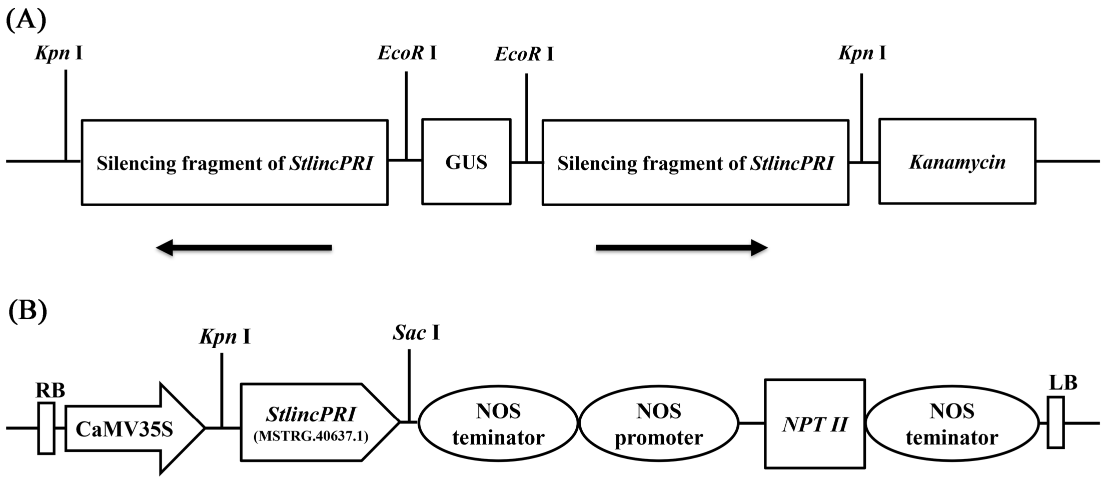

Figure S4. Vector backbones for *StlincPRI* overexpression and silencing. (A) Silencing vector backbone of *StlincPRI*. (B) Overexpression vector backbone of *StlincPRI*

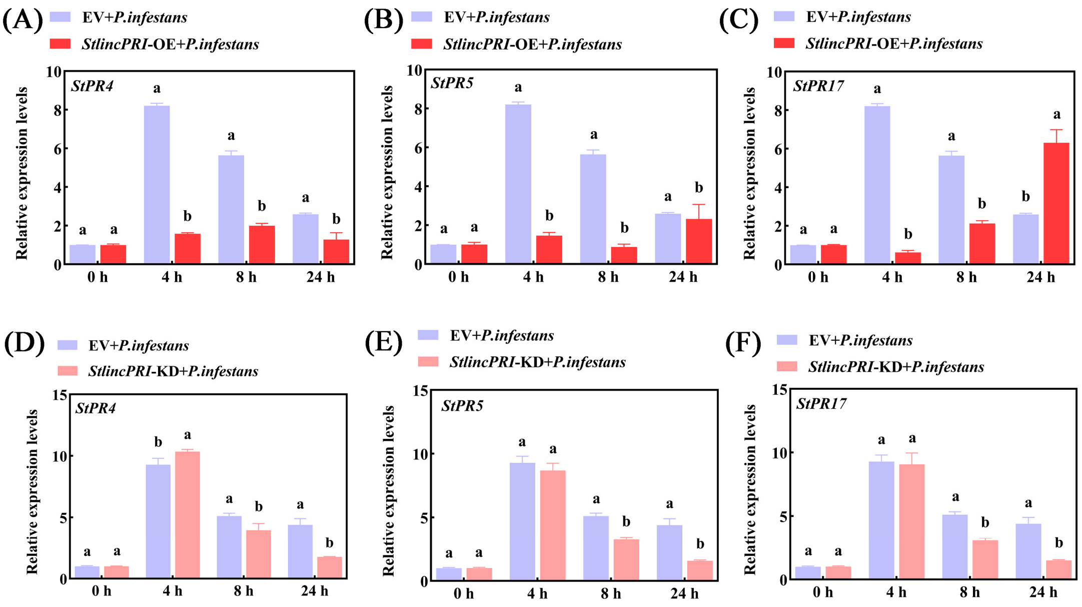

Figure S5. *PR* gene expression following *P. infestans* inoculation. (A–C) Relative expression of *StPR4*, *StPR5*, and *StPR17* in *StlincPRI*-OE leaves and the corresponding EV controls. (D–F) Relative expression of these genes in *StlincPRI*-KD leaves and the corresponding EV controls. Data are means ± SD (n=3) from three independent biological replicates. Each panel was analyzed using ordinary two-way ANOVA with treatment, time, and their interaction, followed by Šídák-adjusted treatment–control comparisons across the four time points. Within each time point in a given panel, different letters indicate a significant treatment–control difference ($P < 0.05$); shared letters indicate no statistically significant difference.

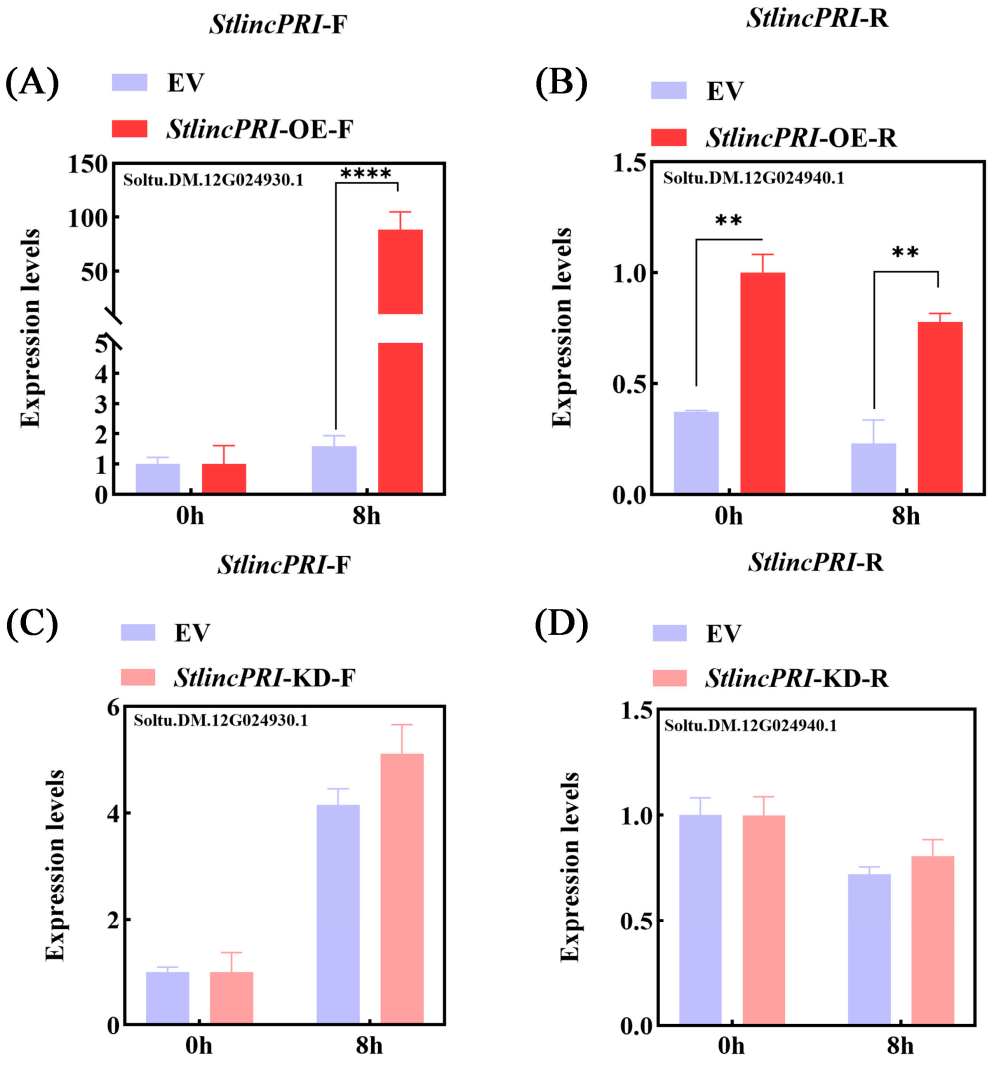

Figure S6. Expression levels of genes flanking *StlincPRI* 1. Soltu.DM.12G024930.1 is the upstream gene, and Soltu.DM.12G024940.1 is the downstream gene. Data are presented as mean ± SD (n=3). Significance was determined by Student's t-test. ***p*<0.01; *****p*<0.0001.
